# Vitamin D – a scoping review for Nordic nutrition recommendations 2023

**DOI:** 10.29219/fnr.v67.10230

**Published:** 2023-11-13

**Authors:** Magritt Brustad, Haakon E. Meyer

**Affiliations:** 1 Department of Community Medicine, University of Tromsø – The Arctic University of Norway, Tromsø, Norway; 2 Department of Community Medicine and Global Health, Institute of Health and Society, University of Oslo, Oslo, Norway; 3 The Public Dental Health Service Competence Centre of Northern Norway (TkNN), Tromsø, Norway; 4 Norwegian Institute of Public Health, Oslo, Norway

**Keywords:** vitamin D, cholecalciferol, ergocalciferol, recommendations, NNR23

## Abstract

Vitamin D is an essential nutrient. Its role in calcium and phosphorous metabolism, and in the development and maintenance of a healthy skeleton is well documented. In addition, there is some evidence for vitamin D decreasing total mortality and cancer mortality modestly, but not cancer incidence. Vitamin D is unique, as both diet and sun induced production in skin are sources to this vitamin. Individual vitamin D status is thus a sum of both sun exposure and dietary intakes. The discovery of vitamin D receptors and the activation of biological active vitamin D in numerous tissues and organs in the body has given support to hypothesis on vitamin D having extra-skeletal functions. The scientific literature on vitamin D and several health outcomes is high in numbers and has been increasing exponentially the last two decades. However, despite this large body of scientific publications and improvement in study quality, vitamin D supplementation has not shown to give additional health benefits when status is in sufficient range (i.e. circulating 25 hydroxyvitamin D >50 nmol/L). Well-designed studies on insufficient or deficient individuals are lacking.

The totality of evidence does not support that increased intake of vitamin D beyond current recommendation will have additional beneficial health effects.

## Popular scientific summary

- Vitamin D has important roles in calcium and phosphorous metabolism, as well as the development and maintenance of a healthy skeleton.
- Deficiency can cause rickets in infants and osteomalacia in adults/older people.
- Vitamin D can be obtained from both dietary sources and via sun exposure.
- Circulating 25-hydroxyvitamin D (25(OH)D) is the most reliable indicator of vitamin D status.
- Vitamin D may slightly reduce overall mortality and cancer mortality, but supplementation may not provide additional health benefits when vitamin D levels are already sufficient.

Vitamin D_3_ (cholecalciferol) is a steroid-like molecule synthesised from 7-dehydrocholesterol in the skin by ultraviolet B (UVB) light from the sun (wavelength between 290 and 315 nm) (1). Humans’ requirement for vitamin D_3_ can be met by exposing the skin to the sun radiation within this wavelength range. However, all the Nordic and Baltic countries (54–71°N) are situated at latitudes where the sun radiation is not sufficient part of the year for vitamin D_3_ production in skin to occur (2). This time of the year is often referred to as the ‘vitamin D winter’. The duration of the vitamin D winter is increasing by increasing latitude.

Food sources of vitamin D_3_ are fish and seafood especially fatty fish like salmon, trout, mackerel, and herring. Egg yolk also contains vitamin D_3_. Some foods contain the metabolite 25-hydroxyvitamin D [25(OH)D], in addition to their cholecalciferol (vitamin D_3_) content (like eggs), but there is no consensus on how to calculate the total contribution of vitamin D in such foods (3). Dairy products (including milk, butter and margarine) are fortified to a various degree in the Nordic countries. During recent years, the use of vitamin D fortification in different foods like oil and plant-based alternatives to milk products has increased, but it differs between countries. More details can be found in Itkonen et al. (4).

Vitamin D_2_ (ergocalciferol) is a form of vitamin D used in some supplements and fortified foods. Some mushrooms like chanterelle are natural sources of D_2._ There is some evidence for D_3_ being more potent than D_2_ in raising vitamin D status in humans; however, inconsistency in results from studies assessing this has been reported (5).

Vitamin D is regarded as a pro-hormone. After entering the body, it is first converted (hydroxylated) to 25-hydroxyvitamin D [25(OH)D] in the liver. Thereafter it is further hydroxylated to the active form of vitamin D, 1,25-dihydroxyvitamin D (calcitriol), predominantly in the kidneys but also in other tissues. Circulating 25(OH)D is considered to be the most reliable biomarker for vitamin D-status in humans as it captures both dietary intake and cutaneous vitamin D-production. Consensus on cut-offs for defining biomarker-levels for ‘sufficient’, ‘insufficient’ and ‘deficient’, has been somehow hard to reach. However, based on available evidence there is a growing agreement that circulating 25(OH)D above 50 nmol/L corresponds to sufficient level, and less than 25–30 nmol/L indicates deficiency (5–8).

Vitamin D deficiency can occur if the diet is devoid of the vitamin and there is little or no exposure to UVB radiation. Infants can develop rickets and adults/elderly people can develop osteomalacia, and for this reason vitamin D is considered an essential micronutrient. The role of vitamin D for curing rickets in children, in mineralisation of bone, and calcium and phosphorous homeostasis is well described (6). Due to the role of vitamin D in securing a healthy skeleton, studies on vitamin D and the prevention of osteoporosis and fractures are many; however, most intervention studies are on vitamin D in combination with calcium.

Possible, so called ‘extra-skeletal’ effects of vitamin D on human health have gained increased research-attention during the last two decades as both the enzymes for activation of vitamin D and the vitamin D receptor are found in numerous tissues and organs in the body. For example, vitamin D is believed to play a role in various organ systems like muscle, brain, pancreatic β cells, and may potentially impact on the cardiovascular as well as the immune system (7). There are indications from e.g. genetic and molecular studies that vitamin D has various extra-skeletal effects (9). Many observational studies have shown associations between vitamin D and a long list of non-skeletal health outcomes and conditions.

The Nordic Nutrition Recommendations (NNR) from 2012 recommendation for vitamin D was based on a systematic literature review by Lamberg-Allardt et al. on quality assessed available evidence (7). This work concluded that there was an overall large heterogeneity in the literature, but conclusive evidence for protective effects on bone health, total mortality, and the risk of falling. It was emphasized that most intervention studies leading to these conclusions reported that intervention with vitamin D combined with calcium and not vitamin D alone, gave these benefits. Due to limited number of high quality randomized controlled trials available, the so-called strength of evidence (SOE) for some of the suggested health outcomes, was weak. Between the former NNR and NNR2023, there has been large increase in total number of scientific studies on vitamin D and health, also with some methodological improvements.

As a basis for setting DRVs, the aim of this scoping review is to describe updated evidence concerning the role of vitamin D on health-related outcomes since the previous version of NNR (NNR2012) (Box 1). Possible effects of vitamin D in the general population are considered, not for specific patient groups.

Box 1. Context and process

- This paper is one of many scoping reviews commissioned as part of the Nordic Nutrition Recommendations 2023 (NNR2023) project (10).
- The papers are included in the extended NNR2023 report but, for transparency, these scoping reviews are also published in Food & Nutrition Research.
- The scoping reviews have been peer reviewed by independent experts in the research field according to the standard procedures of the journal.
- The scoping reviews have also been subjected to public consultations (see report to be published by the NNR2023 project).
- The NNR2023 committee has served as the editorial board.
- While these papers are a main fundament, the NNR2023 committee has the sole responsibility for setting dietary reference values in the NNR2023 project.

## Methods

This review follows the protocol developed within the NNR2023 (10). The sources of evidence used in this scoping review follow the eligibility criteria described in the paper ‘The Nordic Nutrition Recommendations 2022 – Principles and methodologies’ published in Food & Nutrition Research (11).

Due to the very large amount of literature available on vitamin D and health, the literature search strategy was limited to ‘umbrella reviews’ or ‘review of reviews’. This research strategy was decided in dialog with the NNR2023 Committee.

With assistance and advice from the Head Librarian at The University Library, UiT The Arctic University of Norway (Grete Overvåg), the search was developed. The full search string is found in Appendix A. The time period for the search was from January 2011 throughout 22 October 2021.

Proper quality assessment instruments are lacking for umbrella reviews, and an adapted version of AMSTAR 2 (Appendix B) was therefore developed and used in this review. Not all questions in AMSTAR 2 are relevant for evaluating the quality of umbrella reviews. The original AMSTAR 2 consists of 16 questions. Our adapted AMSTAR 2 contained the following original questions: 2,3,4,5,7,8,13,14, and 16 (confer Appendix B for details) as these were considered relevant for umbrella reviews. Question #3 was, however, slightly adapted as it was evaluated as fulfilled if the selection was ‘reported and/or justified’. Thus, ‘explanation for selection of study design for inclusion’ as it is stated in the original # 3 question in the AMSTAR 2 tool, was not required. The #4 (literature search strategy used) was mandatory. Our adapted evaluation tool also included an additional final column where evaluation tool used in the included umbrella reviews was reported. The final grading of quality was based on a discretionary assessment, as the actual AMSTAR scores were not possible to calculate. The results from the included umbrella review were summarized in an evidence table (Supplementary Table 1).

Both the selection and quality assessment were done individually by the two authors and discordance discussed to reach agreements. In addition to the results from the systematic search and selection of umbrella reviews, qualified SRs (qSRs) identified by the NNR2023 Committee (5–7, 12, 13) constituted the source of evidence in this work. We have also consulted the UK Scientific Advisory Committee on Nutrition (SACN) publication from December 2020; i.e. published after the identification of qualified review (2019) by the NNR2023 Committee, entitled *Update of rapid review: Vitamin D and respiratory tract infections* (14).

The remaining qSRs defined by the NNR2023 Committee were not included as final sources, as they were considered too old or newer updated versions were identified in the mentioned systematic search.

This review does not include an assessment of the dose-response relation between vitamin D intakes and circulating 25(OH)D concentrations. This is covered in Appendix 7 of the NNR2023 report (10).

## Physiology

Vitamin D is the generic term for both vitamin D_3_ (cholecalciferol) and vitamin D_2_ (ergocalciferol). They are formed from their respective provitamins ergosterol and 7-dehydrocholesterol (7-DHC) upon UVB radiation exposure and subsequent thermal isomerisation. Vitamin D_2_ differs from vitamin D_3_ in the side chain where it has a double bond between C22 and C23 and an additional methyl group on C24.

### Metabolism

**Fig. 1:**
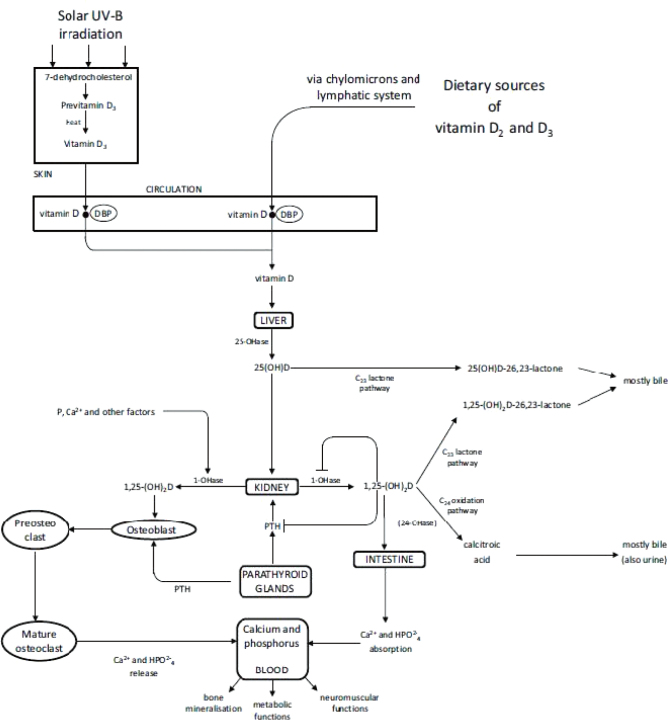
Vitamin D metabolism (6).

The liver rapidly takes up vitamin D formed in the skin or absorbed from the gut where it is hydroxylated to 25(OH)D (Fig. 1). This metabolite is transported in plasma bound to the vitamin D binding protein (also known as the group-specific protein, Gc). The metabolite 25(OH)D is further converted into 1,25-dihydroxyvitamin D (calcitriol) in the kidneys. This is a calcium-regulating hormone that becomes active after binding to a nuclear vitamin D receptor. Together with parathyroid hormone and calcitonin, 1,25-dihydroxyvitamin D ensures that the concentration of calcium and phosphate in the plasma is maintained within narrow limits. Its main function is to stimulate the absorption of calcium from the intestine. In concert with parathyroid hormone, it also stimulates release of calcium from bone thereby increasing the concentration of circulating calcium. Deficiency of vitamin D may result in defective mineralization leading to the development of rickets in children and osteomalacia in adults (7).

The presence of both enzymes for activation of vitamin D and the vitamin D receptors in several tissues, as well as epidemiological and experimental data indicate that vitamin D might also have extra-skeletal roles.

### Uptakes and bioavailability

There is evidence for both increased absorption of vitamin D from oral intakes and higher level of skin production in people with deficient vitamin D status compared to sufficient status (6). There is some evidence that the bioavailability differs between different food sources (6, 7).

Due to low content of vitamin D in breast milk, infants are recommended to receive vitamin D supplementation in all Nordic countries. In the former NNR, the vitamin D recommendation has been equal for children, adolescents, pregnant or lactating women, and adults. Vitamin D status remains stable during pregnancy (5). Due to reduced skin production capacity by age and reduced absorption for elderly, recommendation is higher for those >75 years.

There are some indications in the literature (15) that intake of an equal dose of vitamin D may give different 25(OH)D levels, due to different genetic profiles (polymorphisms). However, the practical implications of this are unclear.

### Assessment of nutrient status

Plasma or serum concentration of 25(OH)D represents total vitamin D from diet and cutaneous synthesis. It is the most reliable biomarker for vitamin D status. However, widespread method-related discrepancies between different laboratories have caused concerns, especially when comparing data from different studies and laboratories. As a response to this problem, several efforts have been made to standardize results across laboratories. This includes the extensive Vitamin D Standardization Program (VDSP), which also has been used to standardize 25(OH)D measurements from population-based studies in the Nordic countries (16).

The liquid-chromatography-tandem-mass-spectrometry (LC-MS/MS) is considered the most valid method for measurement of vitamin D metabolites. The 25(OH)D metabolite has a mean half-life in blood at around 2–3 weeks. Despite laboratory assay challenges, it is the established biomarker for both vitamin D2 and vitamin D3 (6). There is broad consensus that 25(OH)D levels below 25–30 nmol/L indicate vitamin D deficiency (5).

## Dietary intake in Nordic and Baltic countries

In general, data on intakes in Europe has shown increased intakes by increasing latitude. However, there are large variations between the Nordic countries when it comes to level of fortification of vitamin D in foods, which in turn affects the actual intake. Finland is an example of an extensive vitamin D fortification program which has contributed to an improved vitamin D status in the population (4). Supplementation, especially in the form of cod liver oil, is also widely used, especially in Iceland and Norway.

Concerning infants, EU legislations require fortification of infant formula. In accordance with EU legislation, cereal-based products for infants/young children are fortified with vitamin D (4, 17).

The Nordic and Baltic counties are situated within the ‘vitamin D winter window’ where the UVB radiation from the sun is not sufficient for skin to produce vitamin D part of the year (2). This requires oral sources to the vitamin to secure sufficient vitamin D status in the population.

There are vulnerable groups in the population due to low intake or limited sun exposure. Skin pigmentation will also, to some degree, attenuate vitamin D production. At-risk groups include some immigrant groups from Asia and Africa, old people, infants, and teenagers. Strategies have been developed to increase intakes in line with recommendations in these groups (4).

Mean vitamin D intake (excluding supplements) has been reported to be markedly below the recommended intake in all Nordic and Baltic countries except for Finland (18). The higher intake in Finland is in line with their successful voluntary vitamin D fortification policy (18).

Based on selected studies, mean concentration of 25(OH)D seems to be in the sufficient range and approximately similar in the Nordic countries (60–65 nmol/L) among adults, except that older data indicate that the concentrations are lower in Iceland (4). Despite this, a notable proportion of the populations have concentration <50 nmol/L (vitamin D insufficiency), especially during winter. We refer to the Appendix 7 of the NNR2023 report (10) for dose-response discussions.

## Health outcomes relevant for Nordic and Baltic countries

### Results from systematic research of umbrella reviews

**Fig. 2:**
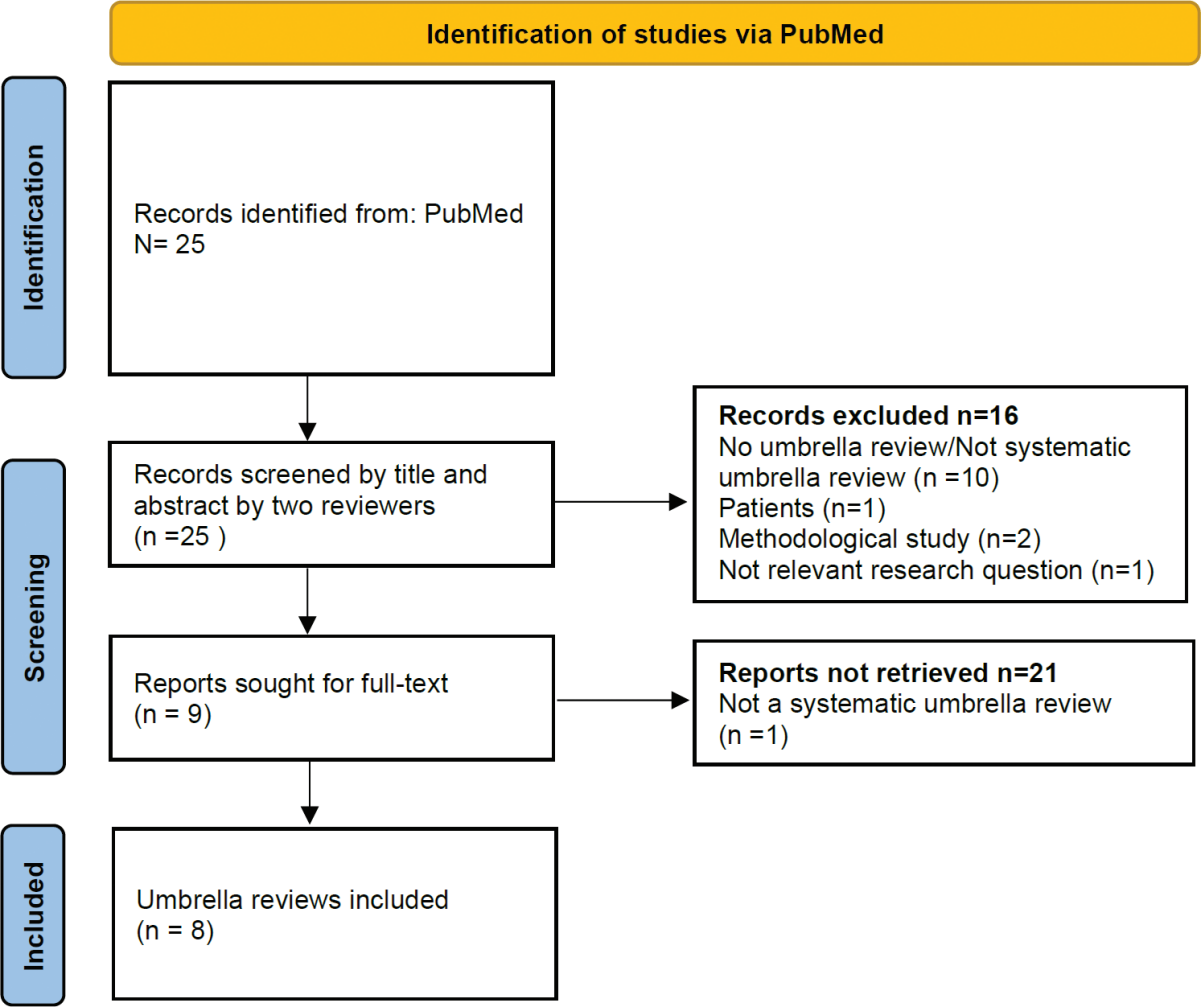
Flow diagram of identification and inclusion of umbrella reviews. Source: Adapted from http://www.prisma-statement.org/.

The selection procedure for umbrella reviews, reasons for exclusions, and final papers identified in the systematic search are all shown in the flow chart in Fig. 2.

The search in PubMed returned 25 hits. After independent assessment by two reviewers (MB and HEM) of title and abstract and discussion of discordances, nine umbrella reviews/review of reviews were selected for further evaluation. The identified papers were quality assessed by use of the adapted AMSTAR 2 checklist (Appendix B). Eight umbrella reviews were evaluated as being of sufficient quality, whereas one paper (19) was excluded because it was not a systematic review.

### Health outcomes in umbrella reviews and qualified systematic reviews

Numerous health outcomes were included in the identified umbrella reviews. They are listed in Supplementary Table 1 (evidence table). Most of the included outcomes or topics in the identified umbrella reviews and qualified reviews were also included in the former NNR2012 process. However, there have been methodological improvements, increased number of studies and published original papers on these outcomes the last decade. New assessed outcomes identified in the umbrella reviews, or the qualified systematic reviews not mentioned in the former NNR2012 process, were: dementia/cognitive disorders, neuropsychological functioning, asthma, and pain. In addition, some of the outcomes mentioned in NNR2012 have now been more elaborated in the recent literature, including infection-related outcomes with specific infections/inflammation conditions as well as some immunomarkers and cancer mortality.

### Conclusive evidence in NNR2012 – is it still valid?

In the systematic review which constituted the basis for the former NNR2012 recommendations on vitamin D by Lamberg-Allardt et al., it was concluded that vitamin D combined with calcium, but not vitamin D alone, reduced the risk of fracture (total fracture and hip fracture) (7).

The results from this present review of the umbrella reviews, confirm the lack of effect of vitamin D supplementation alone (without calcium) on the prevention of fractures in intervention studies. Challenges in the interpretation of the contribution of vitamin D in studies both administering vitamin D and calcium were already mentioned and discussed in the systematic review by Lamberg-Allardt et al. (7). The evidence for vitamin D not having an effect alone (without calcium) on preventing falls and fractures, has now been strengthened.

The recent literature has given increased evidence-weight for a small protective effect of vitamin D on total mortality. Three (20–22) of the identified umbrella reviews report vitamin D preventing total mortality with significant effect estimates around 6% or less. Autier et al. (21) reported that reduced mortality associated with vitamin D supplementation was not modified by concomitant calcium supplementation. The SACN vitamin D and health report from 2016 (5) reports reduced mortality risk for vitamin D and calcium combined.

### Evidence-status for suggested relevant health outcomes

The hypotheses and list of other health outcomes and conditions possibly affected by vitamin D status are, to various degrees, supported by both biological- and observational evidence. However, updated systematic reviews including an increased number of randomized clinical trials (RCTs) have in general not shown clear beneficial preventing effects of vitamin D.

The umbrella reviews identified support a preventive effect on cancer mortality ranging from 12 to 16% reduction in risk estimates (21, 23), but not on cancer incidence.

Biological evidence for vitamin D’s immunomodulatory and anti-inflammatory properties (24) supports the hypothesis on vitamin D having beneficial role on immune responses. In the identified umbrella reviews, we found some evidence supporting a preventive effect of vitamin D on acute respiratory tract infections (ARTI) (25). The *Update of rapid review: Vitamin D and acute respiratory tract infections* by SACN December 2020 (26) aimed at assessing evidence from RCTs on vitamin D and risk of ARTIs published after the 2016 SACN report on Vitamin D and Health (5). The SACN 2020 (26) concluded that vitamin D *may* reduce the risk of respiratory tract infection, but that the size of any potential benefit of vitamin D in reducing acute RTI risk may be small. Further, the SACN 2020 report (26) also concludes that evidence does not support vitamin D supplementation as preventive means for ARTIs, due to large degree of heterogeneity both methodologically with regards to study settings, doses, reporting and assessment outcomes, as well as the fact that RCTs published after 2017 did overall not report preventive effect of vitamin D on ARTIs (5).

Three (20, 27, 28) out of the eight identified umbrella reviews encompassed pregnancy outcome related topics. Strongest support was articulated by Matteussi et al. (20) based on their review on Cochrane systematic reviews where they conclude with ‘some benefits from vitamin D supplementation’ on preterm birth risk and low birth weight with a risk reduction at 64 and 60% respectively. However, this was not supported in the two other identified umbrella reviews (27, 28) mainly due to low quality evidence.

Due to low methodological quality, conclusions could not be made on an effect of vitamin D on cognition and dementia (25, 27, 29).

### Toxicity

Hypercalcemia, bone demineralization, calcification of soft tissue and renal damage are reported as outcomes of acute and chronic exposure to very high vitamin D intakes. Hypercalcemia has been defined as the most accurate endpoint (5). Adverse effects of excess vitamin D were not the topic for any of the identified umbrella reviews; however, some RCTs have reported increased risk for some outcomes under study (falls and fractures) in the intervention group after high dose vitamin D supplementation given as very large bolus doses (5).

The European Food Safety Authority (EFSA) 2016 (6) states that circulating 25(OH)D concentrations above 220 nmol/L may lead to hypercalcemia. The qualified review from EFSA, *Update of the tolerable upper intake level for vitamin D for infants* (12), concludes with upper limit (UL) of 25 μg/day for infants 0–6 months, and a UL of 35 μg/day for infants 6–12 months.

The UL in NNR2012 at an intake of 100 µg/d, was based on the conclusion by EFSA in 2012 (30) and Institute of Medicine (IOM) (31) in 2011. The identified literature for this review, does not provide additional evidence for revising this former UL.

## Requirement and recommended intakes

The potential disease prevention role of vitamin D has gained much attention in the past decade. This attention has resulted in a large increase in original papers as well as reviews. During this decade, the total scientific knowledge in the field has moved upwards in the evidence hierarchy, from observational evidence to increasing number of RCTs, meta-analysis, and systematic reviews.

Overall, the summarized evidence extracted from the large amount of literature shows that as methodological quality increases, the evidence has become weaker for a preventive effect of vitamin D on most outcomes.

There is observational support for associations between vitamin D status and health on various health outcomes. However, the interpretation for associations has challenges due to various biases and confounding. On the other hand, one could argue that RCTs have some methodological limitations in favor for observational studies. This is because RCTs on vitamin D cannot necessarily capture the sufficient long-time exposure of vitamin D relevant for some diseases, the control group is never a ‘non-exposed group’, as well as undefined dose-response curves causing inappropriate designs with regards to intervention-doses and baseline-levels. On the other hand, it is also well known that vitamin D status is associated with other risk factors for disease like physical activity or fish intakes (7). In addition, circulating 25(OH)D might be influenced by disease processes, and reverse causation may occur (5).

### Bone health including falls

Vitamin D’s role in preventing rickets and osteomalacia is well established. In the different revisions on dietary recommendations during the last decades, intakes to control these diseases have not been a pronounced issue when setting recommendations, as preventing these diseases is believed to require only modest doses of vitamin D (5).

Bone health in addition to rickets and osteomalacia was the main determinant for the recommendations in IOM (32) and in the systematic review for the NNR2012 by Lamberg-Allardt et al. (7). The main challenge in interpreting the available literature on these endpoints has been the administration of both calcium and vitamin D in the intervention groups. In Lamberg-Allardt et al. (7), it was concluded that no fracture preventing effect of vitamin D alone has been shown in RCTs. As more studies have been conducted intervening with vitamin D alone, this statement has been strengthened.

Lamberg-Allardt et al. (7) concluded that there was ‘overall fair evidence that vitamin D with calcium is effective in preventing falls in the elderly especially in those with low baseline 25(OH)D concentrations, both community dwelling and in nursing care facilities’. Limited new information was found in the identified umbrella reviews. Mateussi et al. (20) only included systematic reviews already included in Lamberg-Allardt et al. Theodoraout et al. (27) concluded that based on RCTs, vitamin D alone had no preventive effect on falls.

Based on available literature assessed in this review, there is little or no evidence that raising circulating 25(OH)D concentration above 50 nmol/L has any additional bone health impact.

### Non-skeletal health outcomes

Hypothesis on non-skeletal health benefits from increased circulating vitamin D concentrations has caused the exponential increase in research on vitamin D and numerous health outcomes in the last two decades. There is strong biological evidence for vitamin D having a role beyond calcium metabolism and the mineralization of bone. The mechanistic and molecular knowledge about this is increasing quickly. However, randomized controlled trials have to a large extent failed to confirm health benefits of vitamin D supplementation (except for total mortality, and for cancer mortality where the evidence has been strengthened). On the other hand, methodological critique of these RCTs has focused on the fact that most studies have been conducted in subjects with circulating 25(OH)D concentration above 50 nmol/L, i.e. the level identified as sufficient. In addition, high doses of vitamin D have been administered, either at daily, monthly, or even at yearly intervals.

Interestingly, there are indications of effects from supplementations among subgroups with deficient levels of circulating 25(OH)D concentrations. The ethical challenges, however, with conducting RCTs on selected populations at deficient levels are obvious. With regard to setting recommended intakes for vitamin D, this indication of an effect of supplementation in deficient groups supports the aim of avoiding vitamin D deficiency in the population (5). However, the literature in the current review does not support that circulating 25(OH)D concentrations beyond 50 nmol/L is required to achieve sufficient vitamin D health.

### Knowledge gaps

Methodological improvements, a large number of studies and published original papers have increased the total SOE. In particular, the growing number of well-designed RCTs on vitamin D and several outcomes have increased the SOE compared to the evidence status a decade ago. However, identified weaknesses are challenges related to calcium being administered together with vitamin D interventions, few studies conducted on participants with deficient 25(OH)D concentrations, and still lack of well-designed RCTs on some suggested vitamin D related health outcomes. More knowledge on vitamin D status being a *result of,* more than a *cause of* diseases and ill health, could have methodological implications on future study designs. In addition, more knowledge on the direction of the relation between vitamin D and diseases will have implications on the interpretation of available data. The role of vitamin D in inflammation and inflammation-related diseases is interesting. However, based on today’s knowledge the evidence is weak and cannot guide dietary recommendations.

### Implications for recommendations

There is convincing evidence for recommendations to be set to prevent the population from being vitamin D deficient, defined as 25(OH)D <30 nmol/L. There is an increasing body of evidence showing that there is no additional health benefit from increasing the 25(OH)D levels above the suggested sufficient level at around 50 nmol/L.

Based on the totality of present available scientific evidence on vitamin D and health, the overall picture is in line with what was described a decade ago when the NNR2012 was set. The updated evidence does not justify revision of the recommendations. The SOE has increased due to the large research activity within this field. Thus, there is stronger certainty now to conclude that increasing the recommendations will not have an effect in reducing disease risks in the population.
