# Supplementary material for: Vitamin D – a scoping review for Nordic nutrition recommendations 2023
Source: Food Nutr Res. 2023 Nov 13;67:10.29219/fnr.v67.10230. doi: 10.29219/fnr.v67.10230 (PMC10710863; doi:10.29219/fnr.v67.10230)
Supplement: Supplementary file 1 [file FNR-67-10230-s001.docx]

**Table S1 Evidence table with summary of results for the 8 identified umbrella reviews.**

| First authors | \| **Year** \| \| --- \| | Study designs  N=included papers with SRs/MAs |  | Time/  Last updated | Outcomes | Reported risk reduction with vitamin D | Conclusions  *Copied from abstract* | Comments |
| --- | --- | --- | --- | --- | --- | --- | --- | --- | --- |
| Autier et al (ref) | 2017 | Meta-analysis, (RCTs)  N=28 (MAs) |  | 01.01.2013-31.05.2017 | Mortality, CVD, Cancers/neoplasms, adiposity, Glucose metabolism disorders, Respiratory tract infections, Tuberculosis, Asthma, Daily functioning, MS, Pain, Rheumatic conditions, serum biomarkers for inflammation. | All-cause mortality  Cancer mortality. | *Recent meta-analyses reinforce the finding that 10–20 µg per day of vitamin D can reduce all-cause mortality and cancer mortality in middle-aged and older people’*  *‘The main new finding highlighted by this systematic review is that vitamin D supplementation might help to prevent common upper respiratory tract infections and asthma exacerbations.* |  |
| Theodoratou et al (ref) | 2014 | Systematic rev  Meta-analysis of RCTs and observational studies  N=268 |  | -11th October 2013 | Autoimmune diseases, cancer outcomes, cardiovascular outcomes, cognitive disorders, infectious diseases, metabolic disorders, neonatal/infant/child related outcomes, pregnancy related outcomes, skeletal outcomes (including falls), and “other” outcomes | Maternal vitamin D status or supplementation on birth weight and dental caries in children but good quality studies are needed | *Despite a few hundred systematic reviews and meta-analyses, highly convincing evidence of a clear role of vitamin D does not exist for any outcome, but associations with a selection of outcomes are probable* | Excluded studies intervening with vitamin D and calcium |
| Maretzke et al | 2020 | RRs of RCT and cohort studies + single Mendelian RS  N=73 |  | 1st January 2010 -  March/April/May 2019 | Asthma, dementia, cognitive decline, depression, MS, T1DM | Observational data show associations between vitamin D status and the risk of acute respiratory tract infections (ARI), dementia and cognitive decline, and depression.  SRs of RCTs support observational data only for the risk of ARI | *Since several limitations of the included SRs and existing RCTs do not permit definitive conclusions regarding vitamin D and the selected diseases, further high-quality RCTs are warranted.* |  |
| Sluyter et al | 2021 | Meta analysis from:  Case control  Cross sectional  Nested case control  Prospective cohort  RCTs  N=35 |  | -12^th^ May 2020 | Cancer | Observational evidence for cancer incidence, mortality, particularly colon cancer.  Supplementation reduced cancer mortality | *In conclusion, meta-analyses show that, although observational evidence indicates that low vitamin D status is associated with a higher risk of cancer outcomes, randomized trials show that vitamin D supplementation reduces total cancer mortality, but not cancer incidence. However, trials with larger proportions of vitamin D-insufficient participants and longer durations of follow-up, plus adequately powered data on site-specific common cancers, would provide further insight into the evidence base* |  |
| Rejnmark et al | 2017 | Meta analysis on RCTs  N=46 |  | - 1^st^ Dec. 2016 | CVD, blood pressure, type 2 diabetes (T2D), body weight, birth weight, malignant diseases, respiratory tract infections (excluding tuberculosis), depression, and mortality | Mortality,  Respiratory tract infections | *Published RCTs have mostly been performed in populations without low 25OHD levels. The fact that most MAs on results from RCTs did not show a beneficial effect does not disprove the hypothesis suggested by observational findings on adverse health outcomes of low 25OHD levels.* |  |
| Aghajafari et al | 2018 | All study types with and without Meta analysis  N=11 |  | -June 2017 | Alzheimer disease, dementia, cognitive performance | Observational evidence for dementia | *This systematic evaluation of available systematic reviews provided a clearer understanding of the potential link between low serum vitamin D concentrations and dementia. This evaluation also showed that the quality of the available evidence is not optimal because of both the low methodological quality of the reviews and low quality of the original studies. Interpretation of these systematic reviews should therefore be made with care.* |  |
| Mateussi et al | 2017 | Review of Cochrane systematic reviews intervening with vitamin D  N=27 |  | - 4^th^ April 2017 | Prevention for 10 outcomes (17 for treatment, not included here)  Asthma  Fractures  Maternal outcomes  Cancer  Mortality  Bone mineral density in children  Rickets  Infection in children under 5 y  Falls in elderly living in care facilities | Asthma (reduction of severe exacerbations)  Preterm birth risk  Risk of low birth weight  Mortality  Cancer mortality  Rate of falls in elderly living in care facilities | *Based on moderate to high quality of evidence, the Cochrane systematic reviews included here showed that there were some benefits from vitamin D supplementation for pregnant women and asthma patients and no benefits for preventing fractures.* |  |
| Bialy et al | 2020 | Observational  RCTs  N=13 |  | -January 2019 | Preterm birth  Pre-eclampsia  Gestational diabetes  Small for gestational age  Low birth weight  Stillbirth  Caesarean section | Some observational evidence (preterm birth, Pre-eclampsia, gestational diabetes, small for gestational age), but not for RCT ex for low quality study (small for gestational age) | *There is some evidence from SRs of observational studies for associations between vitamin D serum levels and some outcomes; however SRs examining effectiveness from RCTs showed no effect of vitamin D supplementation in pregnancy with the exception of one predefined outcome, which had low quality evidence. Credibility of the evidence in this field is compromised by study limitations (in particular, the possibility of confounding among observational studies), inconsistency, imprecision and potential for reporting and publication biases* |  |
|  |  |  |  |  |  |  |  |  |
