# Supplementary material for: Vitamin D – a scoping review for Nordic nutrition recommendations 2023
Source: Food Nutr Res. 2023 Nov 13;67:10.29219/fnr.v67.10230. doi: 10.29219/fnr.v67.10230 (PMC10710863; doi:10.29219/fnr.v67.10230)
Supplement: Supplementary file 2 [file FNR-67-10230-s002.docx]

**Appendix A – Search terms in PubMed**

With assistance and advice from the Head Librarian at Science and Health Library, UiT The Arctic University of Norway (Grete Overvåg), the following search term was used in PubMed to capture umbrella reviews/review of reviews, time limited to January 2011 throughout October 22end 2021.

"Vitamin D"[Title] AND ((("overview*"[All Fields] OR ("review"[Publication Type] OR "review literature as topic"[MeSH Terms] OR "review"[All Fields]) OR ("metabolism"[MeSH Terms] OR "metabolism"[All Fields] OR "synthesis"[All Fields]) OR ("summaries"[All Fields] OR "summary"[All Fields]) OR ("cochran"[All Fields] OR "cochran s"[All Fields] OR "cochrane"[All Fields] OR "cochrane s"[All Fields]) OR ("analysis"[MeSH Subheading] OR "analysis"[All Fields])) AND ("reviews"[Title] OR "meta-analyses"[Title] OR "articles"[Title] OR "umbrella"[Title])) OR "umbrella review"[Title/Abstract] OR ("meta-review"[Title/Abstract] OR "metareview"[Title/Abstract]))

This search in PubMed gave 25 hits.

After individually assessing title and abstract and discussing discordance, 9 umbrella reviews/review of reviews were selected for AMSTAR 2 evaluation. One of these were excluded because it was not a systematic review. The selected 8 papers were quality assessed by use of an adapted version of AMSTAR II (not all questions in AMSTAR II are relevant for umbrella reviews). The final grading of quality was based on a discretionary assessment as an AMSTAR score was calculated.
