# Supplementary material for: Vitamin D – a scoping review for Nordic nutrition recommendations 2023
Source: Food Nutr Res. 2023 Nov 13;67:10.29219/fnr.v67.10230. doi: 10.29219/fnr.v67.10230 (PMC10710863; doi:10.29219/fnr.v67.10230)
Supplement: Supplementary file 3 [file FNR-67-10230-s003.docx]

**Appendix B. Quality assessment with modified AMSTAR 2**

| Author | 2  Prior established method | 3  Reported selection of study design# | 4  Literature search strategy used | 5  Duplicate selection | 7  List of excluded papers and justification | 8  Adequate details | 13  RoB accounts | 14 Heterogeneity handling | 16  Conflict of interest reported | Quality assessment tools used in the included  reviews |
| --- | --- | --- | --- | --- | --- | --- | --- | --- | --- | --- |
| Autier et al | ? | 1 | 1 | 0 | 0 | 1 | 1 | 1 | 1 | AMSTAR |
| Theodoratou et al | ? | 1 | 1 | 1 | 0 | 1 | 1 | 1 | 1 | Yes, but actual tools not clear |
| Maretzke et al | 1 | 1 | 1 | 1 | 0 | 1 | 1 | 1 | 1 | AMASTAR |
| Sluter et al | 0 | 1 | 1 | 0 | 0 | 1 | 1 | 1 | 1 | AMSTAR |
| Rejnmark et al | 1 | 1 | 1 | 0 | 0 | 1 | 1 | 1 | 1 | AMSTAR |
| Aghahafari et al | 1 | 1 | 1 | 1 | 1 | 1 | 1 | 1 | 1 | AMSTAR |
| Mateussi et al | ? | 1 | 1 | 1 | 1 | 1 | 1 | 1 | 1 | GRADE |
| Bialy et al | ? | 1 | 1 | 1 | 0 | 1 | 1 | 1 | 1 | AMSTAR/  GRADE |
|  |  |  |  |  |  |  |  |  |  |  |

0=no; 1=yes; ?= uncertain; # Reported and/or justified selection (modified from AMSTAR 2)

Critical methodological weaknesses: No 4 is mandatory
